# Optimal transfer learning based nutrient deficiency classification model in ridge gourd (*Luffa acutangula*)

**DOI:** 10.1038/s41598-023-41120-6

**Published:** 2023-08-29

**Authors:** Finney Daniel Shadrach, Gunavathi Kandasamy, S. Neelakandan, T. Bheema Lingaiah

**Affiliations:** 1 Department of Electronics and Communication Engineering, KPR Institute of Engineering and Technology, Coimbatore, India; 2 Department of Electronics and Communication Engineering, PSG College of Technology, Coimbatore, India; 3 Department of Computer Science and Engineering, R.M.K Engineering College, Tiruvallur, India; 4 School of Biomedical Engineering, Jimma Institute of Technology, Jimma, Ethiopia

**Keywords:** Computational biology and bioinformatics, Data processing, Machine learning

## Abstract

The efficient detection of nutrient deficiency and proper fertilizer for that deficiency becomes the critical challenges various farmers face. The family Cucurbitaceae includes members cultivated globally as a source of indigenous medicines, food, and fiber. *Luffa acutangula* (L.) Roxb, generally called Ridge gourd, belongs to the Cucurbitaceae family and is an annual herb originating in several areas of India, particularly in the coastal regions. Nutrient deficiency detection in ridge gourd is essential to improve crop productivity. In agricultural practises, the early identification and categorization of nutrient deficiencies in crops is essential for sustaining optimal growth and production. Addressing these nutrient deficiencies, we applied the Ring Toss Game Optimization with a Deep Transfer Learning-based Nutrient Deficiency Classification (RTGODTL-NDC) to Ridge Gourd (*Luffa acutangula*). This research proposes a new ring toss game optimization with a deep transfer learning-based nutrient deficiency classification (RTGODTL-NDC) method. The RTGODTL-NDC technique uses pre-processing, segmentation, feature extraction, hyperparameter tuning, and classification. The Gabor filter (GF) is mainly used for image pre-processing, and the Adam optimizer with SqueezeNet model is utilized for feature extraction. Finally, the RTGO algorithm with the deep hybrid learning (HDL) model is applied to classify nutrient deficiencies. The suggested framework has the potential to improve crop management practises by allowing for proactive and targeted interventions, which will result in improved agricultural health, production, and resource utilisation. The outcomes represented by the RTGODTL-NDC method have resulted in improved performance. For example, based on accuracy and specificity, the RTGODTL-NDC methodology rendered maximum $acc{u}_{y}$ of 97.16% and specificity of 98.29%. The outcomes show how effective the transfer learning-based model is in identifying nutrient deficits in ridge gourd plants, as seen by its high level of accuracy.

## Introduction

The family Cucurbitaceae contains members cultivated globally as a fiber, food, and medicine source. *Luffa acutangula* (L.) Roxb, a member of the Cucurbitaceae family and more often known as Ridge gourd, was first spotted in several coastal regions of India^1^. The color of the ridge gourd is dark green and contains a tapered end, the pulp is edible after the skin is peeled off, and the pulp will be white. *Luffa acutangula* fruit contains pipecolic acid, carbohydrates, fat, amino acids, protein, carotene, phytin, alanine, cystine, hydroxyproline, glutamic acid, arginine, glycine, tryptophan, leucine, and serine^2^. Its flowers and leaves have flavonoids, and the herb has glucosides and saponins. The seeds have a fixed oil comprising myristic acids, glycerides of palmitic, and stearic. *Luffa acutangula* or Ridge gourd contains higher nutrition and is commonly known as a nutrition powerhouse due to its varied and rich nutrients^3^. It includes niacin, riboflavin, vitamin C, and other vital amino acids. The peptide and charantin presented in this ridge gourd contain insulin regulatory properties and thereby aid in decreasing blood and urine sugar levels^4^. The high fiber in the *Luffa acutangula* will help maintain proper excretory system functioning and healthy digestion. It is also helpful in reducing body heat and makes the skin brighter, a natural detoxifier, thereby aiding in blood purification and strengthening the immune system^5^. The entire plant can be utilized for the treatment of sores and ulcers.

Everyone knows that plants are getting minerals and nutrients for their growth from the soil. The lack of water and nutrients in the earth would harm the proper development of the plant. It is visible as nutrient deficiency symptoms^6^. To enable prompt initiatives for preventing crop loss because of nutrient deficiency and rises in the yield all over the growth period, simultaneously preventing excessive fertilization with harmful ecological consequences, previously, on-site detection and noninvasive nutrient deficiency were needed. The identification of nutrient deficiencies is finding nutrient limitations of crops, namely phosphorous (P), potassium (K), and nitrogen (N) deficiency. Plants need fourteen necessary minerals to grow and complete their life cycle^7^. The availability of plant growth can be different for every component. It can be influenced by crop genetics (crop variety and species), applied fertilizer, soil property, and soil pH. Prior identification of nutrient deficiencies becomes crucial in practice, enabling prompt interference and ignoring irreparable losses. Nutrient deficiency symptoms are evident in the human eyes and frequently appear if plants are severely damaged^8^. NoninvasiveNoninvasive technology, like canopy reflectance sensors or chlorophyll meters, is commonly adopted. But such optical sensors do not straight observe N content presented in plant tissues. Relatively it measures variations in leaf spectral property induced by N deficiency. For the past few years, deep-learning (DL)-related computer vision (CV) issues have been broadly studied^9^. DL can be implemented for agricultural applications, like crop classification, aerial image semantic segmentation, and plant disease recognition, driven by great economic potential, through RGB images^10^. But the dataset has aerial photos or leaves images, and no one tried to identify nutrient deficiencies in sugar beets.

The RTGODTL-NDC model, which is new and relies on deep transfer learning to classify nutrient deficiencies, is introduced in this paper. The presented RTGODTL-NDC model uses a Gabor filter (GF) for noise removal and image segmentation is performed to determine the region of interest (RoI). Besides, Adam optimizer with SqueezeNet model is utilized. Finally, the RTGO algorithm with the hybrid deep learning (HDL) model is applied to classify nutrient deficiencies. The experimental validation of the RTGODTL-NDC model is tested using a dataset comprising different nutrient levels of ridge gourd.

The main contribution of the paper.

- We suggest a new method for nutrient deficiency categorization using deep transfer learning and ring toss games (RTGODTL-NDC).
- Image segmentation and the Gabor filter (GF), typically used for image pre-processing, are combined in instruction to determine the region of interest. For feature extraction, the Adam optimizer with SqueezeNet Perfect is developed.
- Finally, the organization of nutrient inadequacies is performed using the RTGO method with a hybrid deep learning (HDL) model.

## Literature review

Saensuk et al.^11^ identified betaine aldehyde dehydrogenase (LcBADH) from Luffa cylindrica as a gene related to the aroma in sponge gourd. LcBADH has an exon five single nucleotide polymorphism (SNP) (A > G). The BADH enzyme's ability to generate substrate binding spaces depends on the amino acid transition from tyrosine to cysteine. The marker genotype and segregation ratios can be related. Wang et al.^12^ investigate the isolation and utilization of N and carbohydrates in two Asian vegetable crops, the long bean (*Vigna unguiculata* ssp. sesquipedalis (L.) Verdc.) and the angled luffa (*Luffa acutangula* r (L.) Roxb. Since plant biomass is the most nitrogen intake, N-fertilized long bean plants showed increased leaf greenness instead of maintaining mid-reproductive and flower initiation levels. While there has been no change in NUE, shoot biomass, or blade total N, the maximal N input for luffa suggests that there will be more fruits, a more significant total yield, and greener leaves at the mid-reproductive and flower-initiating phases.

Chanda et al.^13^. This work mainly focused on developing an authenticated RP-HPLC technique for standardizing the fruits chosen by the Cucurbitaceae family with the help of cucurbitacin E. The authentication of the RP-HPLC technique has even been executed based on the ICH procedures. This strategy is used for quantitatively predicting cucurbitacin E in plants belonging to the Cucurbitaceae family and its related research. Shaheen et al.^14^ define the updating of a food composition database for international standards. Fifty-four essential foods (KFs) are added to the original 74 foods to obtain the principal analytical values produced component-specifically. Jatav et al.^15^ study has been taking place for assessing genetic diversity by D2 analysis and PCA. A total of 24 bitter gourd genotypes are cultivated in RBD using three replications, including two checks (Pant karela-2 and Pant karela-1). PCA displayed that the first Eigen root contains maximal 26.83% variations. In contrast, the first six principal component axes explained with 84.05% variations utilized for cluster analysis, recommending that the first six main axes were sufficient for explaining the variation in reduced dimensionality.

Mashilo et al.^16^ provide viewpoints on the genetic resources and genetic diversity, breeding progress, and population structure of bottle gourd. In their study, Blanco-Daz et al.^17^ focused on analyzing the different tissues (mesocarp and exocarp) of fruits from 27 morphologically distinct germplasm accessions (13 commercial hybrids and 14 traditional accessions from various countries). In addition, pattern-recognition methods, including dendrogram cluster analysis, distribution, and PCA, are used to compare acquisitions. Ugulu et al.'s^18^ analysis of public health issues focuses on measuring the mean metal concentrations in soil, water, and *Cucurbita maxima* (pumpkin) samples taken from 3 irrigation zones. Using a graphite furnace and flow injection hydride production, the molybdenum (Mo) level in the specimens was measured. Arsenic (As) was determined using the AAS, while selenium (Se) concentrations were determined using the fluorometric technique.

Abbaspour-Gilandeh et al.^19^ used Artificial Neural Networks and Discriminant Analysis to research 13 distinct rice varieties in Iran and categorize them into white rice, brown rice, and paddy. Using Proximal SVM classifiers, Support-vector Machine, and Least Square-SVM, an Expert Learning Model (ELM) created by Mostafaeipour et al.^20^ outperformed Support Vector Regression and other strategies for predicting wheat yield. Accuracy varied from 85.06% to 93% when K-means and SVM were used to categorize five types of rice deficiencies. Dong and others^21^ Deep learning has good consequences in diagnosing micronutrient deficits in maize when associated with other machine learning methods like KNN, SVM, and ANN.

## The proposed model

**Figure 1:**
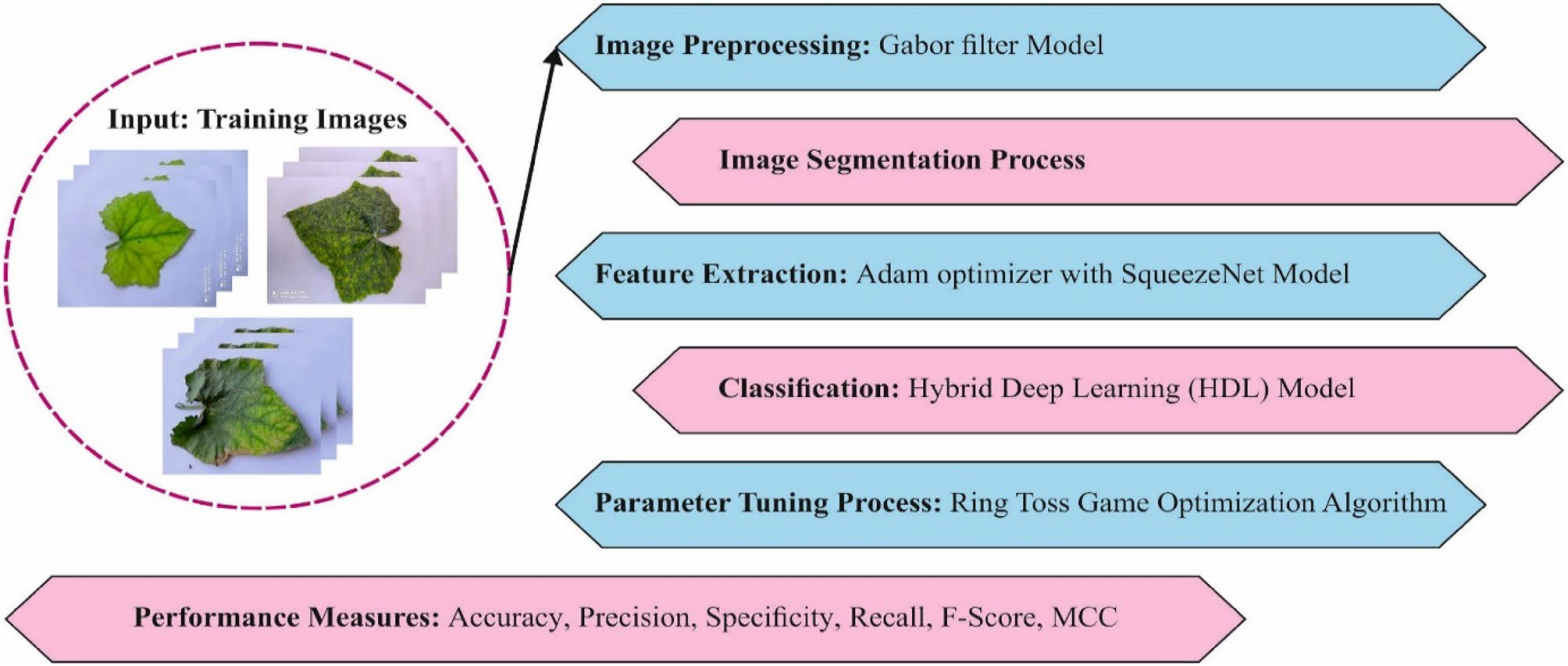
Overall process of proposed RTGODTL-NDC approach.

This research has created a special RTGODTL-NDC algorithm to find and classify various degrees of nutritional inadequacies in ridge gourds. The RTGODTL-NDC technique includes the GF pre-processing, segmentation, SqueezeNet-based feature extraction, Adam-based hyperparameter tuning, HDL-based organization, and RTGO-based hyperparameter optimization procedures. Figure 1 portrays the complete design of the RTGODTL-NDC algorithm.

### Image pre-processing

The GF algorithm is mainly used for picture pre-processing. GF is a linear filter with a unique name^22^. Convolution in the spatial domain is the simple method to perform the filtering method. A picture was convolved with the Gabor elementary performance to acquire the unique appearance. The complex sinusoidal modulated grating of the 2D Gabor function is given in the following.

1 $$g\left(x, y\right)=\frac{1}{2\pi {\omega }^{2}}{\exp}\left\{-\frac{\left({x}^{2}+{y}^{2}\right)}{2{\omega }^{2}}\right\}{\exp}\left[j2\pi \left({u}_{x}+{v}_{y}\right)\right]$$

For ${\omega }_{x}={\omega }_{y},$ $=\omega$ the variables ${\omega }_{x}$ and ${\omega }_{y}$ correspondingly denotes the space constant of the Gaussian envelopes along $x$ and $y$ axes. GF image as ${0}_{h}$ is given below,

2 $${0}_{h}\left(i\left(x, y\right)\right)=\left[i\left(x, y\right)\otimes h\left(x, y\right)\right]$$

### Image segmentation

The segmentation procedure is used to identify the RoI sections of the image after the input images have undergone pre-processing^23^. The idea could first be sharpened using dynamic fuzzy histogram equalization before being segmented to obtain the right ROI. Using feature extraction, valuable data from the section can be retrieved. Segmenting an image is finding similar features inside a picture and then clustering those features together. Data can be broken down into manageable chunks using region- and edge-based approaches. By analyzing the intensity levels of surrounding pixels, region-related segmentation can classify an image into functional or anatomical categories. Using a region-based segmentation technique, the study separates the ROI into regions based on each one's different patterns and textures. Each observation is assigned to a cluster by K-means clustering, which also uses the local mean to produce cluster patterns. Using the entire amount of groups designated by the parameter k, this algorithm looks for clusters in the input dataset. The ideal data point is located using the square of the Euclidean distance. Each data point is categorized into one of the k categories according to the quantified attribute. Data points were divided into sets based on similarities in their characteristics.

### Feature extraction using optimal SqueezeNet model

Adam optimizer with SqueezeNet model is used for feature extraction. Commonly, CNN has a set of hidden layers, an input layer, and a classification layer. Branching and deep layers require sizeable computational power and prolong computation time. This comprises a squeeze convolution layer that includes a 1 × 1 filter and is fed as output to the expanded convolution layer, which can be a mixture of 2 convolution layers correspondingly having 1 × 1 and 3 × 3 filters. The concept was to utilize a 1 × 1 filter for most of the convolution layer and generate a larger activation data pool by postponing the downsampling. Primarily, the downsampling can be performed by setting stride values in the CNN structure to be bigger than 1.

**Figure 2:**
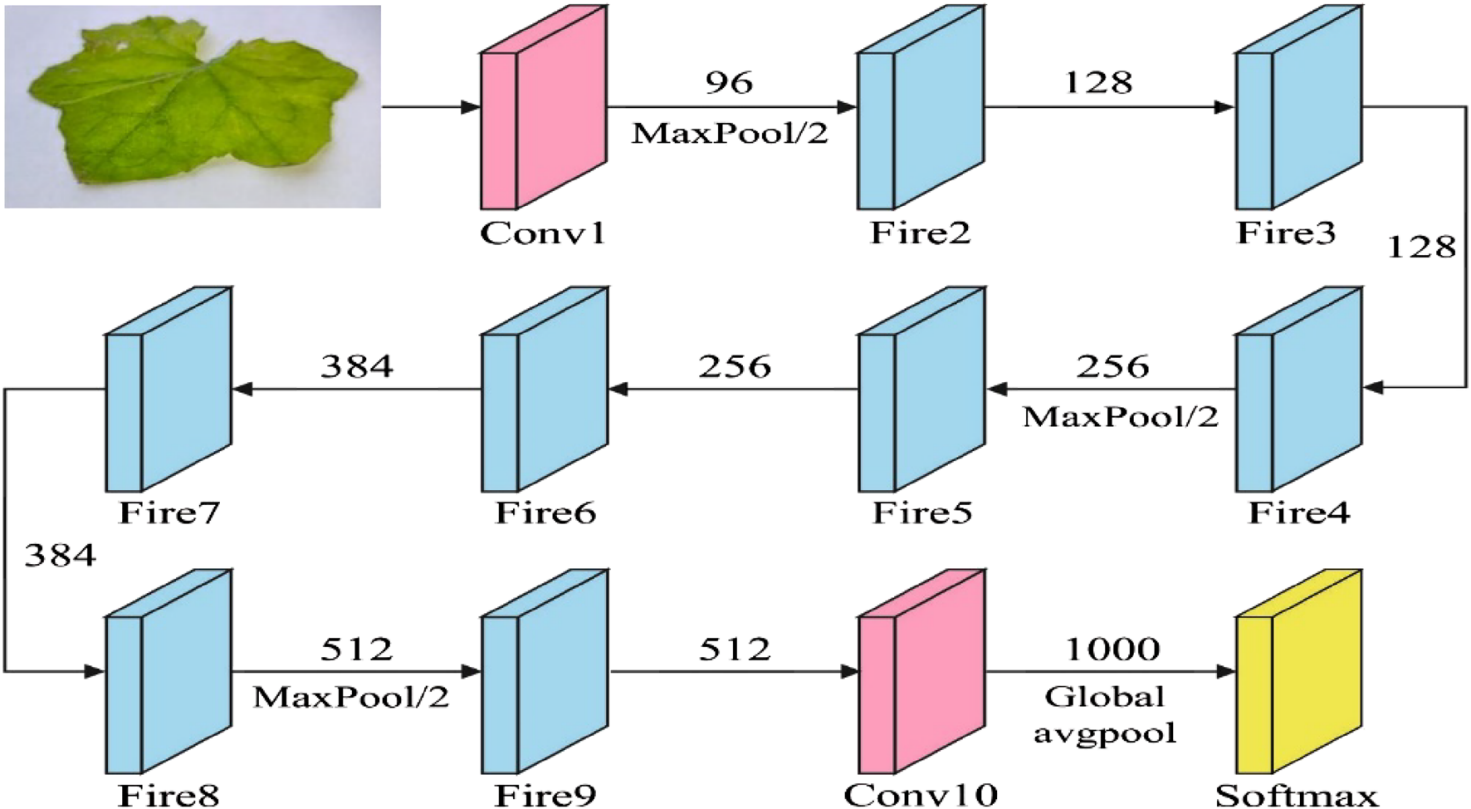
Framework of SqueezeNet.

In SqueezeNet, however, the stride value is retained at one^24^. The pooling layer often follows the activation and convolutional layers. In this study, the SqueezeNet framework is discussed in detail. Figure 2 demonstrates the infrastructure of the SqueezeNet method. A similar CNN related to SqueezeNet is applied for categorizing the waveform for higher precision. To prevent overfitting problems, a particular deep convolution layer is detached from the original network. However, the filter size is improved in the deep convolution layer. The modified network has four fire modules and 39 layers with 42 connections. In the original network, there existed 75 references and 68 layers. It contains eight fire models to extract deep features. But a large filter size is used in the deep convolution layer, like the original SqueezeNet. Then, a relatively larger activation pool is attained. To prevent the overfitting problem, the dropout layer is utilized in this work.

Adam is a first-order optimized technique changing the standard stochastic gradient descent (SGD) procedure^25^. It is iteratively upgrading the NN parameter dependent upon trained data. The SGD maintains a single learning rate for updating every parameter, and the learning rate cannot alter in the trained model. Adam estimates independent adaptive learning rates to distinct parameters by computing the 1st-moment and 2nd-moment estimation of gradients.

**Table:** 

**Algorithm 1:** Pseudocode of Adam optimizer	
$\alpha =0.001,{\beta }_{1}={0}_{ }.9,{\beta }_{2}=0.999,\eta =1{0}^{-8}$(Defaults) ${m}_{0}$ $\leftarrow$ 0 (Initializing ${1}^{st}$ moment vector) ${v}_{0}\leftarrow 0$ (Initializing ${2}^{nd}$ moment vectors) $i\leftarrow 0$ (Initializing step) Whereas ${\Theta }_{i}$ not converged do $i\leftarrow i+1$ ${g}_{i}\leftarrow {\nabla }_{\Theta }{f}_{i}({\Theta }_{i-1})$ (Obtain gradient at step $\dot{i}$) ${m}_{i}\leftarrow {\beta }_{1}\cdot {m}_{i-1}+(1-{\beta }_{1})\cdot {g}_{i}$ (Upgrade biased 1^st^-moment estimate) ${v}_{i}\leftarrow {\beta }_{2}\cdot {v}_{i-1}+(1-{\beta }_{2})\cdot {g}_{i}^{2}$ (Upgrade biased 2^nd^ raw moment estimate) $\widehat{\mathrm{m}}\leftarrow {m}_{i}/(1-{\beta }_{1}^{i})$ (Measure bias‐corrected 1^st^-moment estimate) ${\widehat{v}}_{i}\leftarrow {v}_{i}/(1-{\beta }_{2}^{i})$ (Measure bias‐corrected 2^nd^ raw moment estimate) ${\Theta }_{i}\leftarrow {\Theta }_{i-1}-\alpha \cdot {\widehat{m}}_{i}/({\sqrt{\widehat{v}}}_{t}+\eta )$ (Upgrade parameters) end while return ${\Theta }_{i}$(resultant parameters)	

### Image Classification

Finally, the RTGO algorithm with the HDL model is applied to organize nutrient deficiencies. The study's ultimate goal was to determine an image's hierarchical label. CNN is associated with fine and coarse brands. RNN can be modified to improve productive behavior. Eventually, LSTM worked with RNN through memory cells to train the learning mechanism and demonstrate the progress made in the study for object labeling^26^. CNN generator was utilized to make delicate and coarse labels. Essential modification is done by substituting the final layer of conventional CNN architecture with two layers (Coarse and Fine) and providing supervisory signals to the delicate and coarse categories. The two layers are designed in a parallel or sequential fashion:

3 $$IMAGE\to \left[\left(CONV\to RELU\right)*{N}_{CR}\to POOL\right]*{N}_{CRP}\to FC$$

IMAGE: Input Images

CONV: Convolution Layers

RELU: Rectified Linear Units

POOL: Pooling Layers

$${N}_{CR}$$: up to 5.

$${N}_{CRP}$$ was Large.

$FC$: Contains neurons similar to those in a regular neural network related to the whole strength of the data.

Considering $I$ as the input image, the later convolutional function is determined by Eq. (4)

4 $${C}_{p}=RELU\left(I*{K}_{p}+{b}_{p}\right)$$

In Eq. (4), kernel ${K}_{p}$ employed on input image $I$ and ${b}_{p}$ indicates bias for ${p}^{rh}$ convolutional image.

RELU was a filter-like linear activation function that let efficient suggestion occur by operating undisturbed while setting to zero in the following situations:

5 $$RELU \left(x\right)=\left\{\begin{array}{l}0\,x\le 0\\ x\,x>0\end{array}\right.$$

Consider, ${S}_{p}(x, y)$ be ${p}^{th}$ sampling image afterward employing the pooling function on the activation map ${C}_{p}$. Once filter size $m\times n=2\times 2$, no padding $(P=0)$ and stride $(s=2)$, after AVG pooling can be described by Eq. (6):

6 $${S}_{p}^{1}\left(x,y\right)=\frac{1}{4}{\sum }_{u=0}^{1}{\sum }_{v=0}^{1}{C}_{p}^{1}\left(2x-u, 2y-v\right)$$

RNN can be an artificial neural network where an element builds a structured course between the edges. The input gate is applied for passing clear signals without being interrupted by the output gate to overcome the problem of exploding RNN as given in the following:

7 $${i}_{t}=\sigma \left({W}_{ix}{U}_{xt}+{W}_{ih}{h}_{t-1}+{b}_{i}\right)$$

8 $${g}_{t}=\varphi \left({W}_{gx}{U}_{xt}+{W}_{gh}{h}_{t-1}\right)$$

9 $${f}_{t}=\sigma \left({W}_{fx}{U}_{xt}+{W}_{fh}{h}_{t-1}+{b}_{f}\right)$$

10 $${c}_{t}=\sigma \left({W}_{ox}{U}_{xt}+{W}_{oh}{h}_{t-1}+{b}_{0}\right)$$

11 $${h}_{t}={0}_{t}.\mathrm{ tanh }\left({c}_{t}\right)$$

W and b represent the learning weights and biases required during repeated labeling and learning. Memory cell $({c}_{t})$ is upgraded every time by LSTM to generate innovative labels. Gates allow for the control of every cell's behavior. Creating a hierarchical label for an image was the primary goal of this work. A coarse to delicate pattern is used to measure the label. For acceptable labels, this label may provide useful prediction information.

This work makes use of the RTGO method for the hyperparameter tuning procedure. The RTGO algorithm is mathematically modeled to be implemented on an optimization problem, and the problem variable is described according to the location of every population member^27^. Therefore, every population member is a vector with numerous components equivalent to the number of problem parameters. A population matrix characterizes the population member of the RTGO approach.

12 $$X=\left[\begin{array}{l}{X}_{1}\\ \vdots \\ {X}_{i}\\ \vdots \\ {X}_{N}\end{array}\right]={\left[\begin{array}{ccccc}{x}_{{1,1}}& \dots & {x}_{1,d}& \dots & {x}_{1,m}\\ \vdots & \ddots & \vdots & {\mathinner{\mkern2mu\raise1pt\hbox{.}\mkern2mu \raise4pt\hbox{.}\mkern2mu\raise7pt\hbox{.}\mkern1mu}} & \vdots \\ {x}_{i,1}& \dots & {x}_{i,d}& \dots & {x}_{i,m}\\ \vdots & {\mathinner{\mkern2mu\raise1pt\hbox{.}\mkern2mu \raise4pt\hbox{.}\mkern2mu\raise7pt\hbox{.}\mkern1mu}} & \vdots & \ddots & \vdots \\ {x}_{N,1}& \dots & {x}_{N,d}& \dots & {x}_{N,m}\end{array}\right]}_{N\times m}$$

In Eq. (12), $X$ indicates the population matrix, ${X}_{i}$ is the $i$-$th$ population member, ${x}_{i,d}$ signifies the value of *d*-th parameter for *i*-th members of the population, *N* shows the population count, as well as $m$ implies the problem variable count. The results of estimating the goal function using the population matrix are displayed below.

13 $$OF=[0{F}_{1}\dots 0{F}_{i}\dots 0{F}_{N}{]}_{1\times N}$$

In Eq. (13), OF indicates vector of impartial meaning and $0{F}_{i}$ represents impartial purpose value for $i$-th population. Here, the score bar is installed in the proper location of the search space, whereas the population member provides a better value for the objective function in the following.

14 $$SB={\left[\begin{array}{l}S{B}_{1}\\ \vdots \\ S{B}_{i}\\ \vdots \\ S{B}_{{N}_{SB}}\end{array}\right]}_{{N}_{SB}\times m}$$

Equation (14), $SB$ indicates the matrix of location of the score bar, $S{B}_{i}$ specifies the location of $i$-$th$ score bars in the search space, and ${N}_{SB}$ denotes the score bar count that is equivalent to 10% of members of the population. Then, the throwing of the ring toward the score bar is modeled. Every circle is thrown randomly toward the bar, expressed in the subsequent calculation.

15 $$F=round\left(1+r\right)$$

16 $$d{x}_{i,d}=\left\{\begin{array}{ll}r(s{b}_{i,d}-F{x}_{i,d}),& 0{F}_{S{B}_{i}}<0{F}_{i}\\ r({x}_{i,d}-Fs{b}_{i,d}),& else\end{array}\right.$$

17 $${x}_{i,d}^{new}={x}_{i,d}+d{x}_{i,d}$$

18 $${X}_{i}=\left\{\begin{array}{ll}{X}_{i}^{new},& 0{F}_{i}^{new}<0{F}_{i}\\ {X}_{i},& else\end{array}\right.$$

Equations (15) to (18), $d{x}_{i,d}$ designates the displacement value for $i$-$th$ members of population in $d$-$th$ dimension, $s{b}_{i,cl}$ denotes the $d$-$th$ dimension of score bar location, $0{F}_{S{B}_{i}}$ indicates the objective function of $i$-$th$ score bar, ${x}_{i,d}^{new}$ represents the newly suggested location for $i$-$th$ members of population in $d$-$th$ dimension, $0{F}_{i}^{new}$ denotes the impartial purpose for newly suggested position of $i$-$th$ members of population, and $r$ displays an random value within $[{0,1}]$. Updating members of the population is reiterated based on Eqs. (13) to (18) until the stopping criteria are achieved. Afterward the completion of the iteration, the appropriate quasi‐optimum solution attained using the presented method is available.

The RTGO algorithm derives a fitness function (FF) for exhibiting the enriched performance of the organization. It defines a positive numeral to distinguish the enhanced version of the candidate explanation. In the study, the reduction of classifier error rate can be viewed as the FF in the following. The error rate in the best description is lower, whereas the error rate in the worst explanation is higher.

19 $$\begin{aligned}&fitness\left({x}_{i}\right)=ClassifierErrorRate\left({x}_{i}\right)\\ &=\frac{number\, of\, misclassified\, samples}{Total\, number\, of\, samples}*100\end{aligned}$$

### Results and discussion

**Figure 3:**
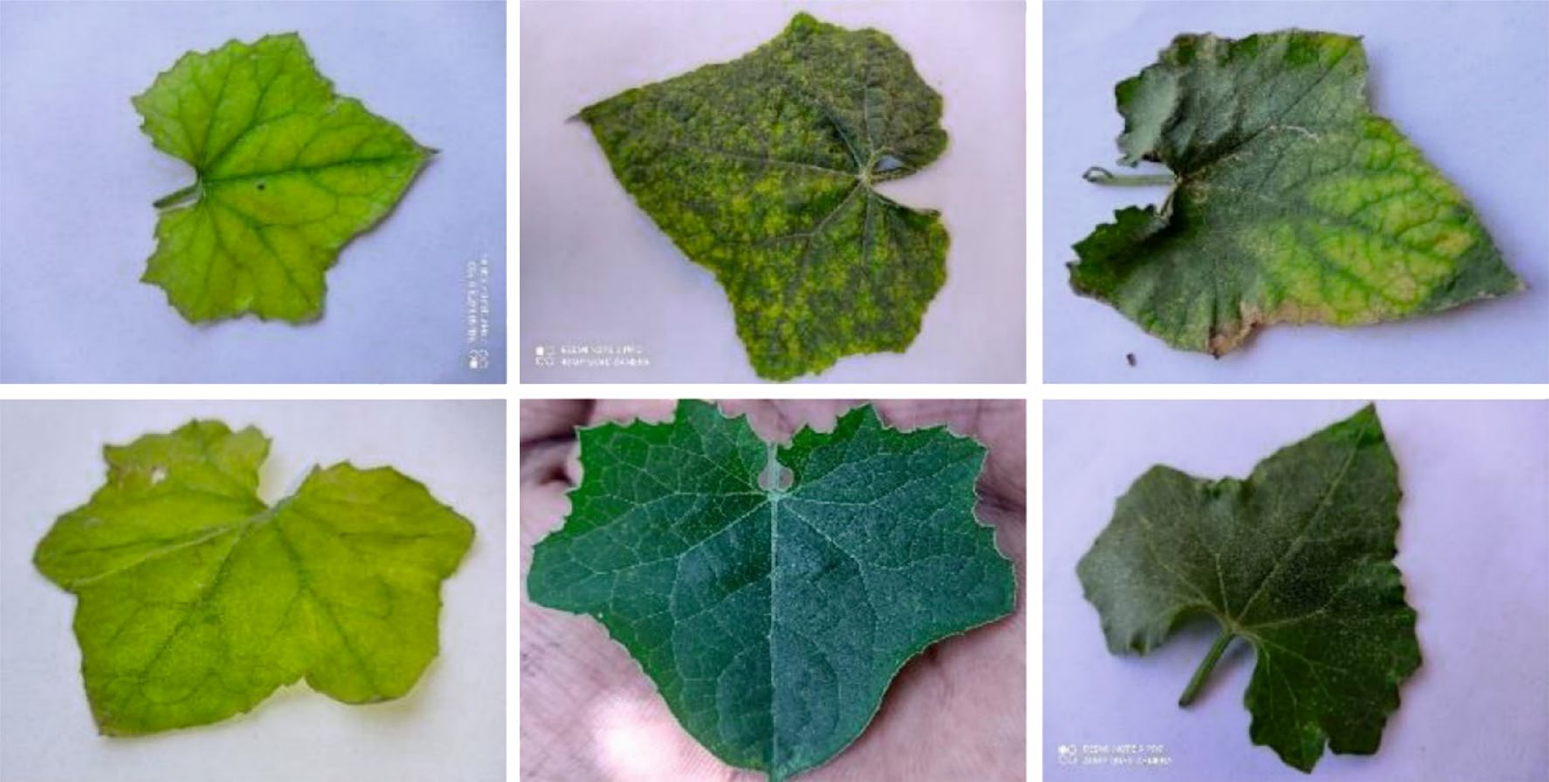
Example images.

The nutrient deficiency detection performance of the RTGODTL-NDC technique can be tested using a rigid gourd dataset comprising 12,700 samples. According to Table 1, the dataset consists of six class labels. In Fig. 3, a few examples of photographs are shown.

**Table 1:** Dataset specifics.

Class	Description	No. of examples
1	Nitrogen deficient	2259
2	Phosphorus deficient	2250
3	Potassium deficient	1923
4	Calcium deficient	1910
5	Iron deficient	2216
6	No deficiency	2142
Entire Number of Examples		12,700

**Figure 4:**
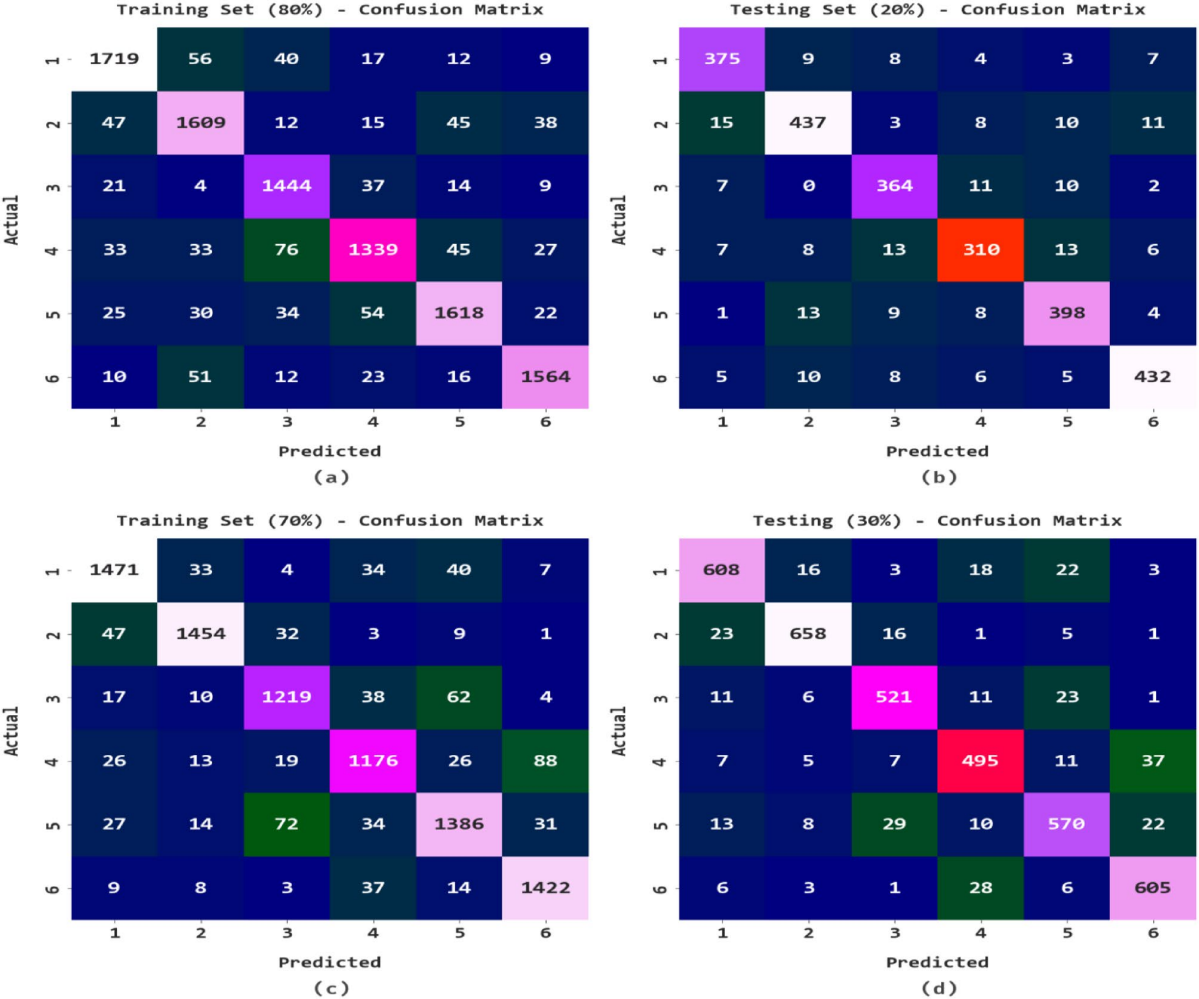
RTGODTL-NDC technique confusion matrices (**a**) 80% of TR data, (**b**) 20% of TS data, (**c**) 70% of TR data, and (**d**) 30% of TS data.

The confusion matrices generated by the RTGODTL-NDC model on the used dataset are shown in Fig. 4. The results showed that the RTGODTL-NDC model performed superiorly in all respects. When the RTGODTL-NDC method was used to analyze 80% of the TR dataset, it was discovered that 1719 samples, among other classifications, belonged to class 1, 1609 samples, class 2, 1444 samples, class 3, 1339 samples, class 4, 1618 samples, and class 6. The RTGODTL-NDC technique effectively assigned 375 samples to class 1, 437 samples to class 2, 364, 310 examples to class 4, 398 samples to class 5, and 432 examples to class 6 using just 20% of the TS data. The RTGODTL-NDC model used 70% of TR data to categorize samples as either 1 (1471), 2 (1454), 3 (1219), 4 (1176), 5 (1386), or 6 (1422). With 608 samples placed in the class, 30% of the TS dataset has been categorized. 1, 658 samples were classified 2, 521 samples 3, 495 samples 4, 570 samples into Classes 5, and 605 samples into Classes 6.

Table 2 presents the comprehensive nutrient deficiency classification outcomes of the RTGODTL-NDC method with 80% of TR data and 20% of TS data.

**Table 2:** Analysis of the RTGODTL-NDC method's outcomes using unique class labels for TR/TS data with an 80:20 ratio.

Labels	Accuracy	Precision	Recall	Specificity	F-Score	MCC
Training set (80%)						
1	97.34	92.67	92.77	98.36	92.72	91.09
2	96.74	90.24	91.11	97.93	90.67	88.70
3	97.45	89.25	94.44	97.98	91.77	90.31
4	96.46	90.17	86.22	98.30	88.15	86.10
5	97.08	92.46	90.75	98.42	91.59	89.83
6	97.86	93.71	93.32	98.76	93.51	92.23
Average	97.16	91.41	91.43	98.29	91.40	89.71
Testing set (20%)						
1	97.40	91.46	92.36	98.36	91.91	90.37
2	96.57	91.61	90.29	98.05	90.95	88.84
3	97.20	89.88	92.39	98.09	91.11	89.47
4	96.69	89.34	86.83	98.31	88.07	86.16
5	97.01	90.66	91.92	98.05	91.28	89.48
6	97.48	93.51	92.70	98.55	93.10	91.56
Average	97.06	91.08	91.08	98.24	91.07	89.31

**Figure 5:**
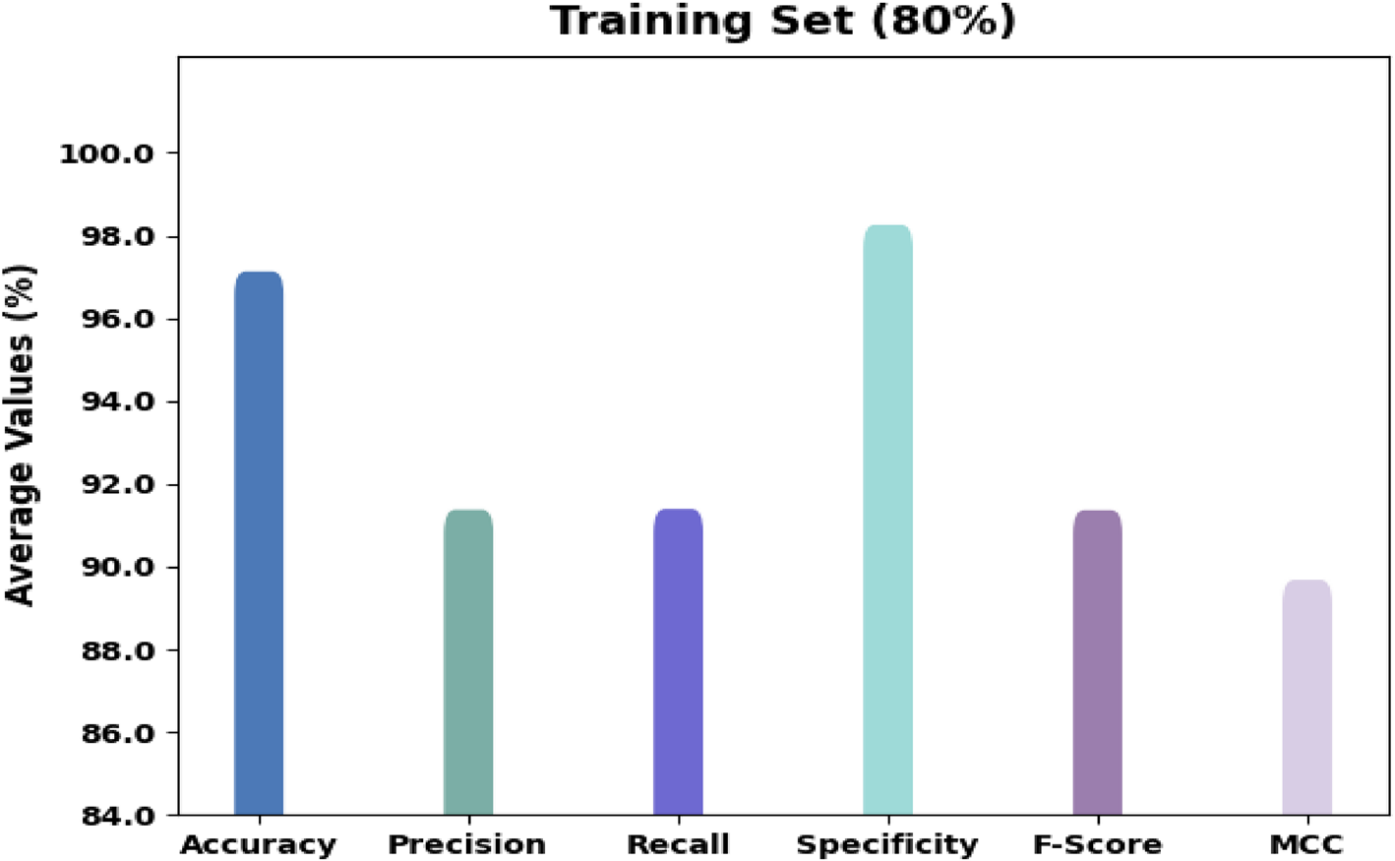
Under 80% of the TR data, the RTGODTL-NDC approach's average analysis.

Figure 5 exhibits the organization outcomes of the RTGODTL-NDC model on 80% of TR data. The consequences implied the RTGODTL-NDC approach had detected all the classes accurately. For example, the RTGODTL-NDC approach has seen images in class 1 with $acc{u}_{y}$ of 97.34%, $pre{c}_{n}$ of 92.67%, $rec{a}_{l}$ of 92.77%, $spe{c}_{y}$ of 98.36%, ${F}_{score}$ of 92.72%, and MCC of 91.09%. In contrast, the RTGODTL-NDC method has recognized images under class 3 with $acc{u}_{y}$ of 96.74%, $pre{c}_{n}$ of 90.24%, $rec{a}_{l}$ of 91.11%, $spe{c}_{y}$ of 97.93%, ${F}_{score}$ of 90.67%, and MCC of 88.70%. Likewise, the RTGODTL-NDC algorithm has standard images under class 2 with $acc{u}_{y}$ of 97.45%, $pre{c}_{n}$ of 89.25%, $rec{a}_{l}$ of 94.44%, $spe{c}_{y}$ of 97.98%, ${F}_{score}$ of 91.77%, and MCC of 90.31%.

**Figure 6:**
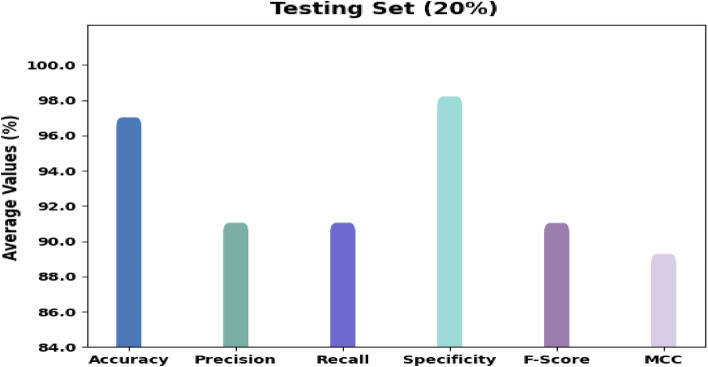
RTGODTL-NDC technique average analysis under 20% of TS data.

Figure 6 displays the organizational consequences of the RTGODTL-NDC technique on 20% of TS data. The outcomes designated the RTGODTL-NDC method have accepted all the classes accurately. For sample, the RTGODTL-NDC method has recognized images under class 1 with $acc{u}_{y}$ of 97.40%, $pre{c}_{n}$ of 91.46%, $rec{a}_{l}$ of 92.36%, $spe{c}_{y}$ of 98.36%, ${F}_{score}$ of 91.91%, and MCC of 90.37%. conversely, the RTGODTL-NDC method has recognized images under class 2 with $acc{u}_{y}$ of 96.57%, $pre{c}_{n}$ of 91.61%, $rec{a}_{l}$ of 90.29%, $spe{c}_{y}$ of 98.05%, ${F}_{score}$ of 90.95%, and MCC of 88.84%. Likewise, the RTGODTL-NDC technique has recognized images under class 3 with $acc{u}_{y}$ of 97.20%, $pre{c}_{n}$ of 89.88%, $rec{a}_{l}$ of 92.39%, $spe{c}_{y}$ of 98.09%, ${F}_{score}$ of 91.11%, and MCC of 89.47%.

Table 3 displays the findings of the RTGODTL-NDC approach's thorough classification of nutritional deficit using 70% TR and 30% TS data.

**Table 3:** RTGODTL-NDC technique with dissimilar class labels under 70:30 TR/TS data outcome analysis.

Labels	Accuracy	Precision	Recall	Specificity	F-Score	MCC
Training set (70%)						
1	97.26	92.11	92.57	98.27	92.34	90.67
2	98.09	94.91	94.05	98.94	94.48	93.32
3	97.06	90.36	90.30	98.28	90.33	88.60
4	96.42	88.96	87.24	98.06	88.09	85.99
5	96.30	90.18	88.62	97.94	89.39	87.15
6	97.73	91.56	95.24	98.23	93.37	92.02
Average	97.14	91.35	91.34	98.29	91.33	89.63
Testing set (30%)						
1	96.80	91.02	90.75	98.09	90.88	88.94
2	97.80	94.54	93.47	98.78	94.00	92.65
3	97.17	90.29	90.92	98.27	90.61	88.94
4	96.46	87.92	88.08	97.91	88.00	85.92
5	96.09	89.48	87.42	97.88	88.44	86.10
6	97.17	90.43	93.22	97.98	91.81	90.11
Average	96.91	90.62	90.64	98.15	90.62	88.78

**Figure 7:**
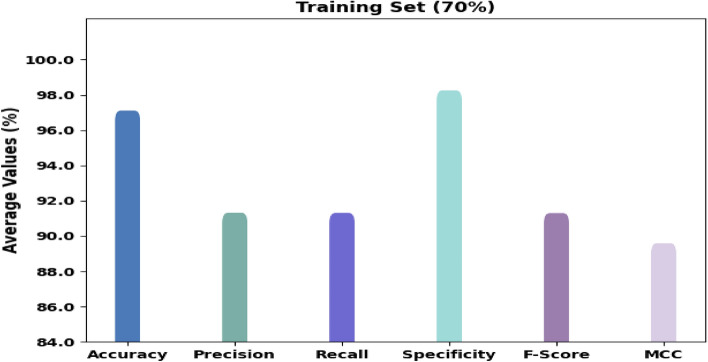
Average analysis of RTGODTL-NDC method under 70% of TR data.

Figure 7 shows the classification outcomes of the RTGODTL-NDC method on 70% of TR data. The consequences denoted the RTGODTL-NDC approach identified all the classes accurately. For example, the RTGODTL-NDC method has recognized images under class 1 with $acc{u}_{y}$ of 97.26%, $pre{c}_{n}$ of 92.11%, $rec{a}_{l}$ of 92.57%, $spe{c}_{y}$ of 98.27%, ${F}_{score}$ of 92.34%, and MCC of 90.67%. In contrast, the RTGODTL-NDC technique has identified images under class 2 with $acc{u}_{y}$ of 98.09%, $pre{c}_{n}$ of 94.91%, $rec{a}_{l}$ of 94.05%, $spe{c}_{y}$ of 98.94%, ${F}_{score}$ of 94.48%, and MCC of 93.32%. In addition, the RTGODTL-NDC algorithm has recognized images under class 3 with $acc{u}_{y}$ of 97.06%, $pre{c}_{n}$ of 90.36%, $rec{a}_{l}$ of 90.30%, $spe{c}_{y}$ of 98.28%, ${F}_{score}$ of 90.33%, and MCC of 88.60%.

**Figure 8:**
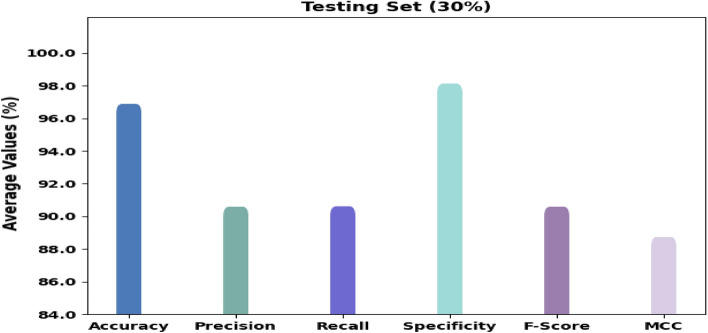
Average analysis of RTGODTL-NDC method under 30% of TS data.

Figure 8 displays the classification outcomes of the RTGODTL-NDC method on 30% of TS data. The consequences denoted the RTGODTL-NDC method identified all the classes accurately. For instance, the RTGODTL-NDC technique placed images in class 1 with $acc{u}_{y}$ of 96.80%, $pre{c}_{n}$ of 91.02%, $rec{a}_{l}$ of 90.75%, $spe{c}_{y}$ of 98.09%, ${F}_{score}$ of 90.88%, and MCC of 88.94%. Then, the RTGODTL-NDC algorithm identified images under class 2 with $acc{u}_{y}$ of 97.80%, $pre{c}_{n}$ of 94.54%, $rec{a}_{l}$ of 93.47%, $spe{c}_{y}$ of 98.78%, ${F}_{score}$ of 94%, and MCC of 92.65%. Additionally, the RTGODTL-NDC technique has recognized images under class 3 with 97.17%, $pre{c}_{n}$ of 90.29%, $rec{a}_{l}$ of 90.92%, $spe{c}_{y}$ of 98.27%, ${F}_{score}$ of 90.61%, and MCC of 88.94%.

**Figure 9:**
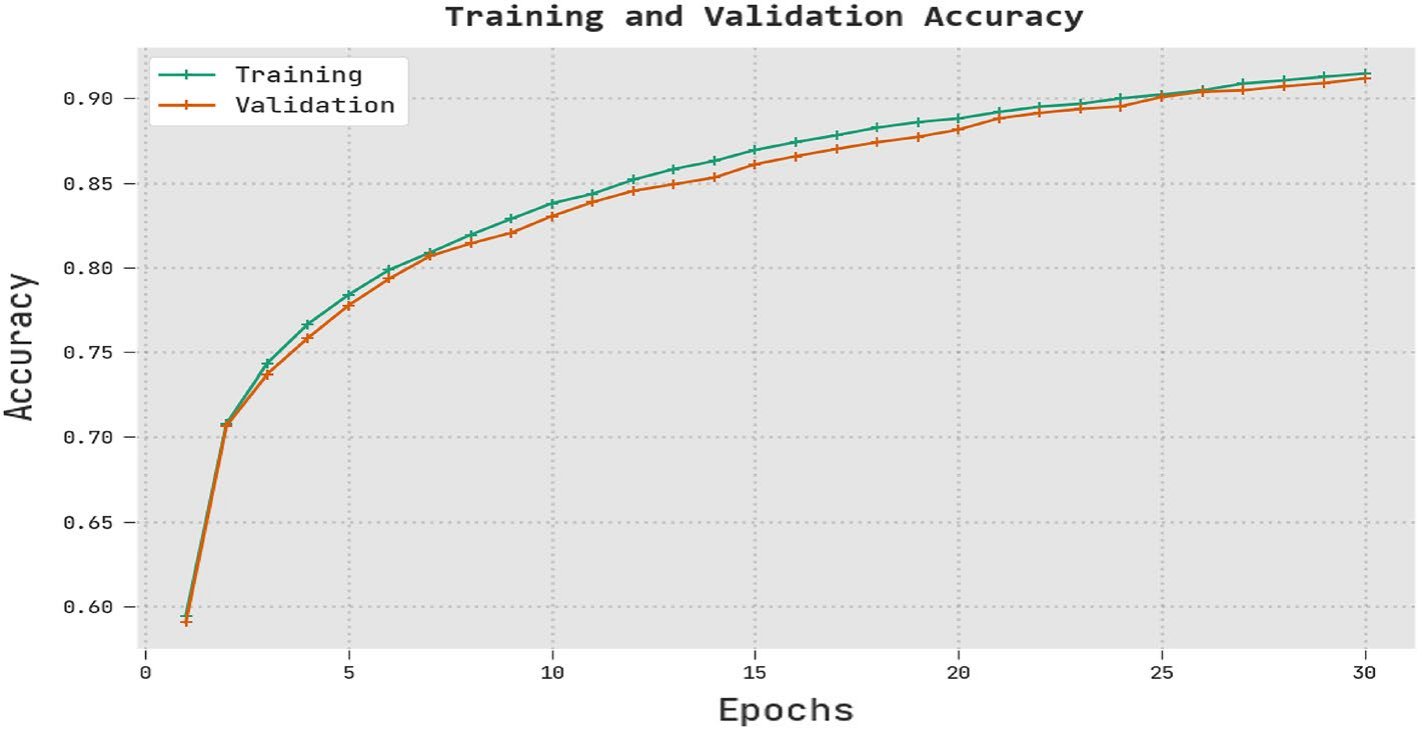
Analysis of the RTGODTL-NDC method using TRA and VLA.

The RTGODTL-NDC approach's training accuracy and validation accuracy on test data are shown in Fig. 9. According to the results of the experiments. The RTGODTL-NDC approach generated the highest TRA and VLA values. The VLA feels like a step up from the TRA.

**Figure 10:**
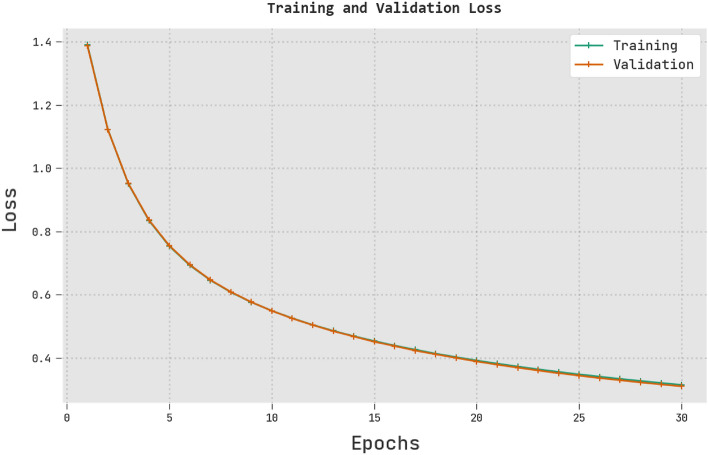
Analysis of the RTGODTL-NDC method's TRL and VLL.

Figure 10 displays the training and validation loss generated by the RTGODTL-NDC technique on test data. The experiment's findings showed low TRL and VLL values for the RTGODTL-NDC method. TRL, in particular, outperforms VLL.

**Figure 11:**
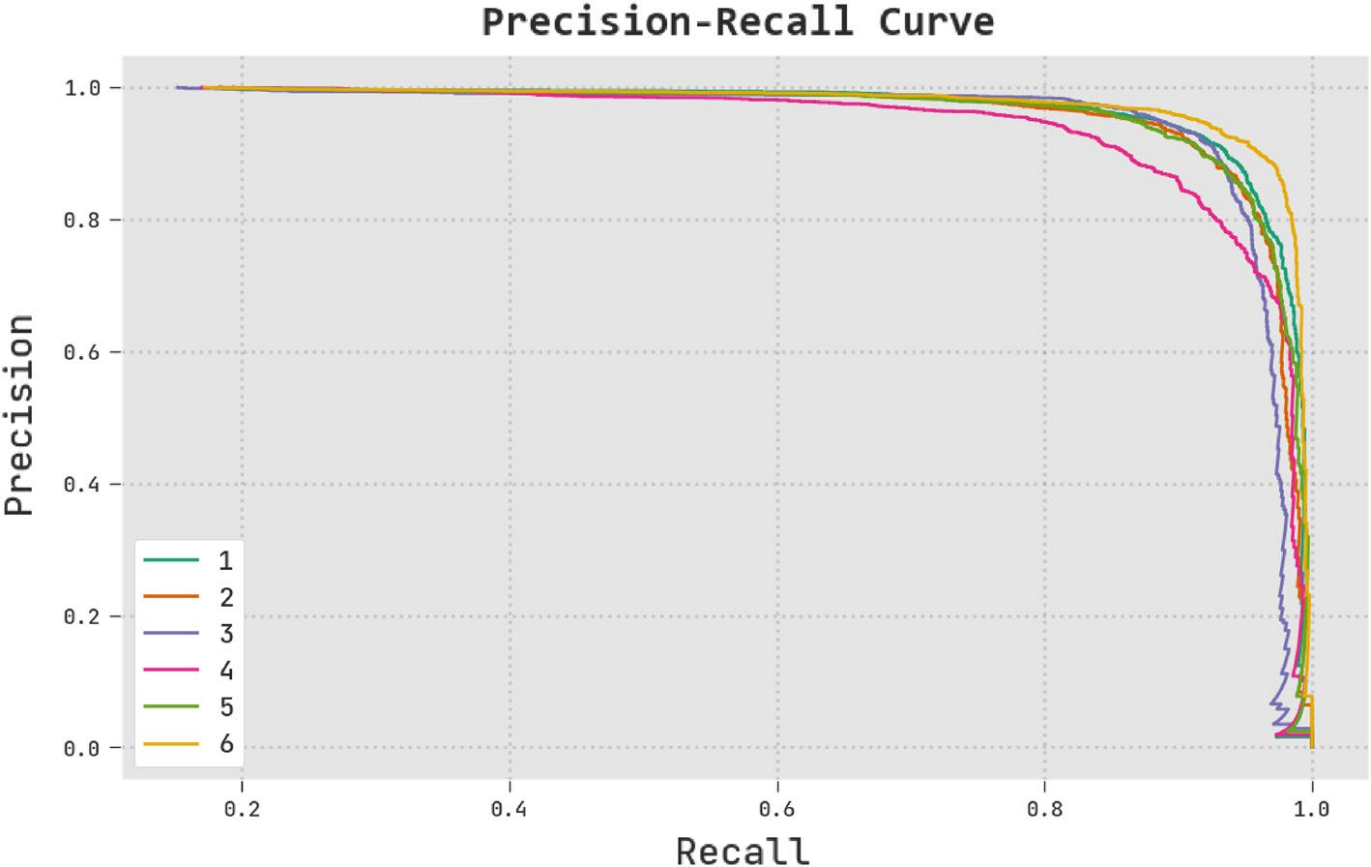
Analysis of RTGODTL-NDC's precision and recall.

The RTGODTL-NDC technique on the test dataset is analysed in terms of precision-recall in Fig. 11. The precision-recall values have increased for all classes using the RTGODTL-NDC approach, as seen in the image.

**Figure 12:**
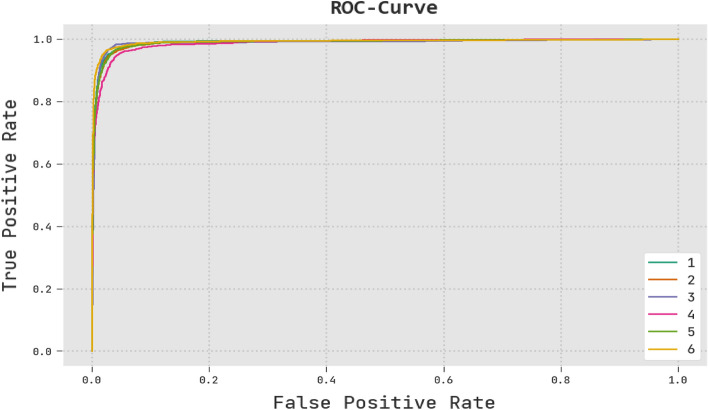
Analysis of the RTGODTL-NDC method's ROC curve.

Figure 12 shows a quick ROC analysis of the RTGODTL-NDC method on the test dataset. According to the results, the RTGODTL-NDC algorithm has proven to be capable of classifying multiple classes on the test dataset.

Table 4 provides a complete correlation of the RTGODTL-NDC methodology with current techniques. The findings show that the RTGODTL-NDC model performs better than other existing methods.

**Table 4:** Analysis of the RTGODTL-NDC approach in comparison to existing algorithms.

Methods	Accuracy	Sensitivity	Specificity	F1 Score
RTGODTL-NDC	97.16	91.43	98.29	91.40
Support vector machine model	92.48	91.17	93.45	90.45
K-nearest neighbour model	92.88	91.04	93.66	91.03
Decision tree model	81.24	79.73	81.49	81.33
Neural network model	95.43	90.26	96.31	91.19
Ensemble model	96.28	91.37	96.43	91.32

**Figure 13:**
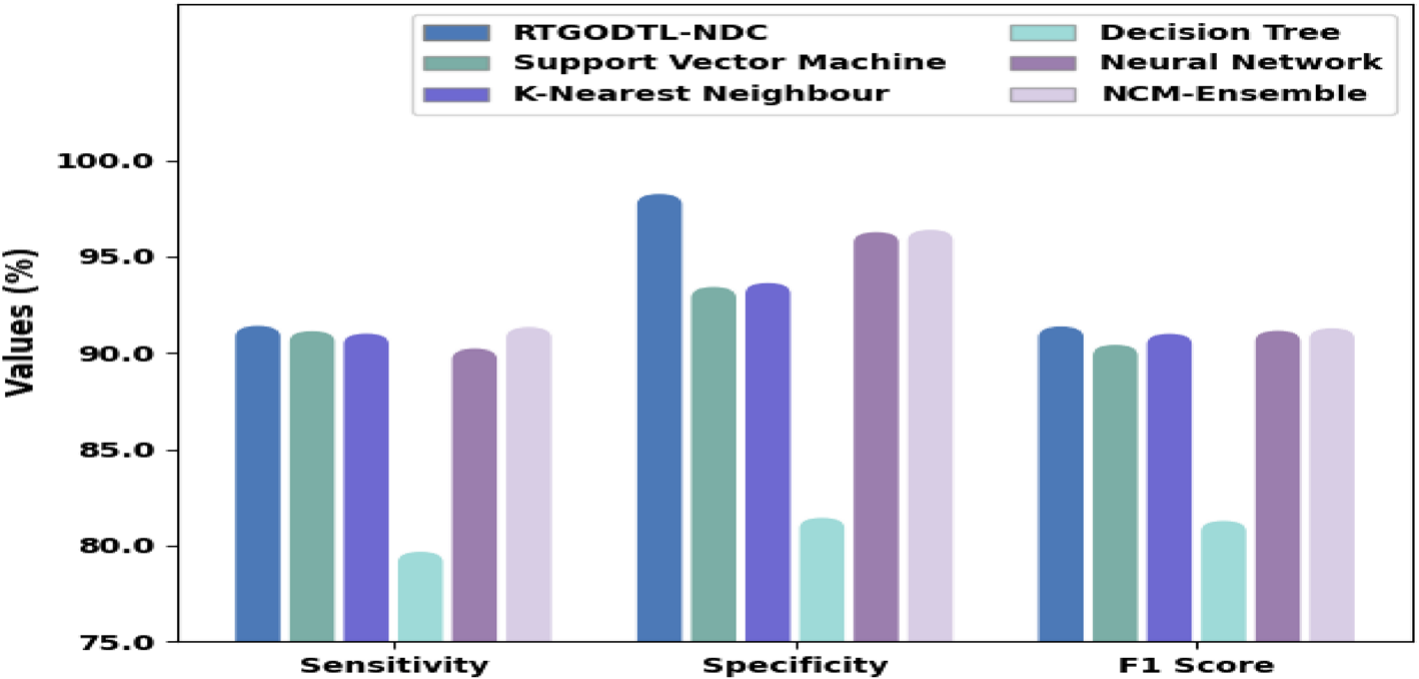
RTGODTL-NDC technique comparison with existing algorithms.

Figure 13 exhibits a relative consequence study of the RTGODTL-NDC model with other approaches in terms of $sen{s}_{y}$, $spe{c}_{y}$, $F{1}_{score}$ an $acc{u}_{y}$ d The consequences signified that the RTGODTL-NDC model had caused improved performance. For instance, based on $sen{s}_{y}$, the RTGODTL-NDC model has provided higher $sen{s}_{y}$ of 97.16% whereas the SVM, KNN, DT, NN, and NCM-ensemble models have accomplished lower $sen{s}_{y}$ of 92.48%, 92.88%, 81.24%, 95.43%, and 96.28% correspondingly. At the same period, based on $spe{c}_{y}$, the RTGODTL-NDC method has rendered higher $spe{c}_{y}$ of 98.29% whereas the SVM, KNN, DT, NN, and NCM-ensemble methods have established lower $spe{c}_{y}$ of 93.45%, 93.66%, 81.49%, 96.31%, and 96.43% correspondingly. Simultaneously, based on $F{1}_{score}$, the RTGODTL-NDC algorithm has provided higher $F{1}_{score}$ of 91.40% whereas the SVM, KNN, DT, NN, and NCM-ensemble models have accomplished lower $F{1}_{score}$ of 90.45%, 91.03%, 81.33%, 91.19%, and 91.32% correspondingly.

**Figure 14:**
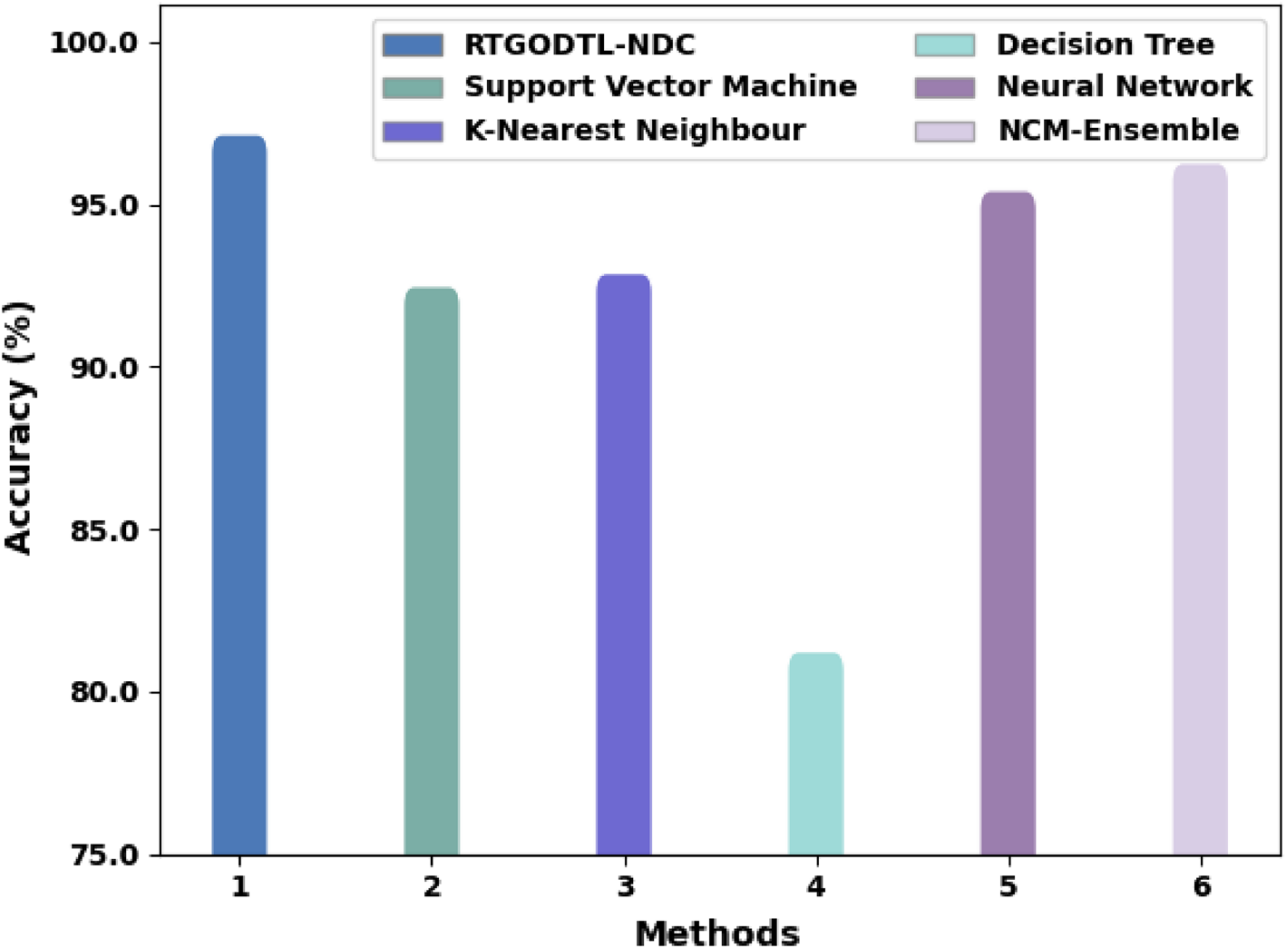
$Acc{u}_{y}$ analysis of RTGODTL-NDC approach with existing algorithms.

A comparison of the RTGODTL-NDC method and other ways in $acc{u}_{y}$ standings are shown in detail in Fig. 14. The outcomes represented by the RTGODTL-NDC method have resulted in improved performance. For example, based on $acc{u}_{y}$, the RTGODTL-NDC methodology rendered maximum $acc{u}_{y}$ of 97.16% while the SVM, KNN, DT, NN, and NCM-ensemble techniques have displayed lower $acc{u}_{y}$ of 92.48%, 92.88%, 81.24%, 95.43%, and 96.28% correspondingly. As a result, the RTGODTL-NDC approach performs better than previous models at detecting nutrient deficiencies.

### Limitations of RTGODTL-NDC model

The focus of this investigation is Ridge Gourd (*Luffa acutangula*) and its nutritional deficiencies. The proposed model discoveries and enhancements may not be applicable to other plant or crop species. Due to differences in symptom manifestation, leaf structure, and development patterns, the effectiveness of the strategy based on deep transfer learning may vary among plants. While the technique based on deep transfer learning shows promise for diagnosing nutritional deficiencies, it is essential to recognise that visual symptoms alone may not always provide an accurate picture of nutrient deficiencies. Variations in soil composition, pH levels, and nutrient assimilation can also influence plant health. These additional variables, which may affect the accuracy of nutritional insufficiency identification, are not taken into account in the study.

### Ethical approval

We confirm that our research does not include a poll in which real human participants are asked to provide their opinions, or animal data used to justify our findings.

## Conclusion

This work proposed a unique RTGODTL-NDC approach for identifying and categorizing different levels of nutrient deficits in ridge gourds. The segmentation, hyperparameter tuning, feature extraction, pre-processing, and organization steps of the RTGODTL-NDC approach are all performed. GF technique is mainly utilized for image pre-processing, and image segmentation is done to identify the RoI portions of the image. For feature extraction purposes, Adam optimizer with SqueezeNet model is utilized. Finally, the RTGO algorithm with the HDL model is applied to organize nutrient deficiencies. A dataset with varying ridge gourd nutrient levels can be used to demonstrate the experimental validity of the RTGODTL-NDC technique. The SVM, KNN, DT, NN, and NCM-ensemble strategies showed lower accuracy of 92.48%, 92.88%, 81.24%, 95.43%, and 96.28%, respectively, whereas the RTGODTL-NDC methodology produced the highest accuracy of 97.16%. As a result, the RTGODTL-NDC approach performs better than previous models at detecting nutrient deficiencies. The results of the experiments show that the RTGODTL-NDC approach performs significantly better than other DL models. Field studies must evaluate the transfer learning model's real-world effectiveness and generalizability. The model's training data may not fully capture how soil, weather, and agricultural practices affect ridge gourd nutrient deficits. In the upcoming years, the detection performance of the RTGODTL-NDC method will be extended to the deep instance segmentation process. A decision support system incorporating the nutrient deficiency categorization model can offer in-the-moment guidance on fertilizer recommendations, nutrient management techniques, and crop-specific actions. Optimised ring toss game and nutrient deficiency classification may be incorporated into a comprehensive decision support system in order to enhance precision agricultural practises. This would help farmers improve their crop-growing processes and increase agricultural output.

## Data Availability

The corresponding author will provide the datasets used and analyzed during the current work upon reasonable request.
